# Zirconia-Toughened Alumina (ZTA) Nanoceramics with a Gradient Microstructure: A Comparative Study of ZTA Ceramics with Fibrous and Granular Morphology

**DOI:** 10.3390/mi14091681

**Published:** 2023-08-28

**Authors:** Eszter Bódis, Zoltán Károly

**Affiliations:** Institute of Materials and Environmental Chemistry, Research Centre for Natural Sciences, Magyar tudósok krt. 2, H-1117 Budapest, Hungary; karoly.zoltan@ttk.hu

**Keywords:** Zirconia-toughened alumina, ZTA, nanofiber, nanograins, ceramic composites, porosity gradient, temperature gradient, SPS, HAp

## Abstract

ZrO_2_-toughened Al_2_O_3_ (ZTA) ceramic composites with a porosity gradient and with improved mechanical properties have a wide range of possible applications. We fabricated nanofibrous and nanogranular Y-ZTA and Ce-ZTA composites with a gradient microstructure by creating a temperature gradient during SPS sintering, with the use of asymmetric graphite tool arrangement (ASY). In this study, we examined the morphology effect of the starting materials on the sintering process and on the final microstructure, as well as the mechanical properties of the composites. A large temperature difference was established for both the granular and fibrous samples fabricated in the ASY configuration, which resulted in gradient porosity along the ceramics bodies: the upper part of the ceramics showed a highly porous fine microstructure, while the opposite side was highly densified. The final microstructure of the composites can be tailored by varying the morphology of the starting ceramics or the graphite configuration. A highly porous skeleton-like structure was formed by sintering fibres in the ASY configuration, whereas the granular precursors resulted in a much less porous composite. The microstructure affected the mechanical properties of the composite. Improved hardness and more than 50% higher compression strength were obtained for the granular Ce-ZTA samples as compared to the fibrous sample. Gradient porosity with fibrous or granular morphology promotes the penetration of bioactive nanosized hydroxyapatite (HAp) into the pore structure. Fibrous ZTA absorbs HAp more effectively due to its higher porosity as well as its bimodal pore structure.

## 1. Introduction

Owing to their low density, high-thermal resistance and chemical stability, porous ceramics are widely used as filters, catalyst supports, sound absorbing materials, etc. They could also be used for biomedical applications: bone and teeth repair or regeneration [1,2].

Due to the requirements of particular applications, several manufacturing methods have been elaborated to produce porous structures, including gel-casting [3], the organic foaming agent technique [4], freeze casting [5,6] and the pore-forming agent method [7], just to mention a few. However, one of the simplest, one-step fabrication processes is partial sintering [1], which is a conventional technique for making porous ceramics and it has been substantially developed in recent years. Apart from its simplicity, this procedure does not require special additives, such as a pore-forming agent or special equipment. As sintering progresses, the particles in the compacted powder are bonded by surface diffusion or evaporation–condensation mechanisms enhanced by heat and pressure during sintering. As a result, a homogeneous porous structure is formed when sintering is terminated before full densification. The pore framework or structure is also determined by the neck formation mechanism between the particles of the starting materials [7]. In general, the porous ceramic body developed by partial sintering usually consists of particles, but several papers reported on fibres as starting materials [8,9,10]. With short or continuous fibres, a three-dimensional network of interconnected pores can also be produced as a bond formed between the fibres during sintering [10,11].

One of the main drawbacks of porous ceramics is their inferior mechanical properties as compared to the corresponding dense materials. Thus, improving the mechanical properties of porous ceramics is an important issue for their reliability in practical applications. Ceramic materials with a gradient porosity structure may provide a solution for this apparent contradiction. In these ceramics, porosity shows a continuous change along the axial direction of the bulk material. Porosity gradient materials are a special type of functional graded materials (FGMs), showing a gradient in terms of a certain properties such as microstructure and/or composition along the body. As a result, the gradient structure exhibits multifunctional properties, e.g., in porosity gradient materials, the highly porous part provides high-absorption capacity (e.g., for the bone cells), while the opposite part with high density offers enhanced mechanical stability for the sample. FGMs are considered attractive as biomaterials and as permanent skeletal replacement implants too [2,12,13,14], since bones also have a functionally graded structure from the cortical bone at the surface towards the inner cancellous bone.

Several procedures were developed to fabricate graded porous structures, including templating [15,16], direct foaming [17] or freeze casting [18]. These methods consist of several steps and require additives or components for the development of the final microstructure. The formation of gradient microstructure by partial sintering is also a viable option if a significant temperature gradient can be created within the ceramic body during heat treatment. Several studies have reported that a temperature gradient can be developed either in the axial or radial direction [19] during SPS. A specially designed graphite tool [20], or a simple asymmetric graphite configuration [21,22,23], can produce inhomogeneous current distribution in the axial direction, and in turn, a temperature gradient in the samples [20,24,25,26]. The latter results in a gradient in porosity distribution [25].

Spark plasma sintering (SPS) utilizes Joule heating and/or electric discharge to consolidate the powder. Several studies focused on the above-mentioned phenomena with respect to powder compaction during SPS, but only a few papers addressed the effect of the morphology of the raw powder on the heating mechanisms. Vidyuk et al. [27] reported that particles with higher width-to-length ratios showed a greater tendency to melt at the contact points. Chaim [28] assumed that the evolved electric discharge depends on the morphology of the starting particles. Preston et al. [29] studied austenitic stainless steels with spherical, plate-like and flake morphology during SPS using asymmetric powder compaction. They found that powders with higher aspect ratios led to larger thermal gradients. Therefore, Joule heating or sparks can be produced in various ways at the connecting points, according to the various morphology of the starting materials.

In this study, we fabricated nanosized zirconia–toughened alumina (ZTA) composites using two different stabilizing agents. CeO_2_ was applied in 16 mol% and Y_2_O_3_ in 3 mol% to stabilize the ZrO_2_-toughened Al_2_O_3_, hereinafter referred to as Ce-ZTA and Y-ZTA, respectively. In addition, the morphology of the ceramic composites was also varied and compared with the use of granular and fibrous starting materials. The samples were consolidated under the same SPS conditions (uniaxial pressure, temperature, and graphite configuration). We investigated: (1) the effects of different morphology of the starting materials on the formation of temperature gradient in the sample during SPS; (2) the microstructure; and (3) the mechanical properties of the sintered composites. Furthermore, in this study, we also anticipated the possible use of these ceramics in biomedical applications. Therefore, we studied the impregnability of the developing gradient pore structure. Bioactive nano-hydroxyapatite (HAp) was impregnated into the porous structure and then the microstructure of the composites was studied.

## 2. Materials and Methods

### 2.1. Synthesis of Ceramic Nanofibres and Nanoparticles

Ce-ZTA and Y-ZTA ceramic fibres and particles were synthesized to serve as raw materials for the sintering tests. Fibres were made by electrospinning, while the sol-gel method was employed for the synthesis of ZTA nanoparticles.

The following precursors were used for the synthesis: aluminium nitrate nonahydrate [Al(NO_3_)_3_·9H_2_O] (Szkarabeusz Kft., Budapest, Hungary, 99.0%), zirconyl (IV) chloride hydrate (ZrOCl_2_·H_2_O) (Szkarabeusz Kft., Budapest, Hungary, 99.0%), cerium (III) nitrate hexahydrate (Ce(NO_3_)_3_·6H_2_O) (Sigma-Aldrich Kft., Budapest, Hungary; 99.0%), and yttrium (III) nitrate hexahydrate (Y(NO_3_)_3_·6H_2_O) (Sigma-Aldrich Kft., Budapest, Hungary; 99.0%). The wight ratio of Al_2_O_3_:ZrO_2_ was invariably 80:20. To obtain the ideal tetragonal crystal structure for ZrO_2_, we used 16 mol % CeO_2_ and 3 mol% Y_2_O_3_ as stabilization additives for Ce-ZTA and Y-ZTA, respectively.

The preparation method of ZTA fibres is detailed elsewhere [23]. Briefly, we dissolved the appropriate salts for Ce-ZTA and Y-ZTA in distilled water. To obtain solutions suitable for fibre spinning, we used poly(vinylpyrrolidone) (PVP) with a molecular weight of 1.3 × 10^6^ g/mol as auxiliary material, dissolved in ethanol (Molar Chemicals, Budapest, Hungary, 99%) at a concentration of 25 wt%. We added the PVP-ethanol solution to the ZTA precursor solutions in a ratio of 1:1, and stirred it to adjust the required viscosity for fibre spinning. The appropriate solutions were subsequently injected from a plastic syringe through a stainless steel needle, with an inner diameter of 1 mm, at a constant flow rate of 15 cm^3^ h^−1^ with an Aitecs (Vilnius, Lithuania) SEP-10 syringe pump. A direct current power supply (MA2000 NT 35/P, Budapest, Hungary) of 35 kV was connected to the needle. The distance was 20 cm between the tip of the needle. The raw nanofibers were heat-treated at 1200 °C for 1 h in an atmosphere of air. The average fibre diameters were 530 ± 23 and 520 ± 21 nm for Y-ZTA and Ce-ZTA composite fibres, respectively, determined by SEM image analysis in an earlier paper [23].

The synthesis of Y-ZTA and Ce-ZTA nanopowders was based on the sol-gel method. The precursors were the same as for the fibres. The precursor salts were dissolved in 200 mL of distilled water corresponding to the final 80:20 weight ratio of Al_2_O_3_ and ZrO_2_. For the tetragonal ZrO_2_, the stabilization agent was the same amount as for fibres. The Ce-ZTA and Y-ZTA mixed salt solution was precipitated with the addition of 0.3 M ammonium hydroxide dropwise until the solution reached pH 10 and a white gel was formed. The gel was filtered and washed with distilled water until chlorine ions (Cl^−^) could not be detected with a 0.03 M silver nitrate (AgNO_3_) solution. Finally, the gel was dried in an oven at 90 °C in air for 24 h, then calcined at 1200 °C for 1 h. The average particle size was 190 and 200 nm for Y-ZTA and Ce-ZTA, respectively, according to SEM image analysis (Figure S1).

### 2.2. Synthesis of Bioactive Hydroxyapatite

HAp was synthesized by wet precipitation. In total, 0.3 M of calcium nitrate 4-hydrate [Ca(NO_3_)_2_·4H_2_O] and 0.5 M of diammonium phosphate [(NH_4_)_2_HPO_4_] were dissolved in distilled water as calcium and phosphate sources, respectively, purchased from Szkarabeusz Laboratórium Kft. in analytical grade. The pH value of the reaction solution was maintained at 9.5 by adding a 25% NH_4_OH (Reanal Laborvegyszer Kereskedelmi Kft., Budapest, Hungary) solution. HAp synthesis was performed at 60 °C with a total stirring time of 8 h [30]. The HAp precipitates were washed by distilled water until pH 7 was reached. The as-received HAp was filtered and dried for analysis by phase composition and structure by XRD (Figure S2). However, for impregnation, we used unfiltered and aqueous HAp in 16 V/V% concentration to facilitate its absorption into the ZTA pore structure. The morphological analysis of the synthesized HAp powder is reported in [30].

### 2.3. Synthesis of Porous Ceramics Composites

The fibrous and granular ZTA samples were consolidated with an SPS machine (HPD25, FCT System GmbH, Rauenstein, Germany) under vacuum at two different temperatures (1200 °C and 1300 °C) for 4 min. Both the heating and cooling rate were set to 100 °C/min. We sintered the samples under a moderate uniaxial pressure of 6 MPa in order to obtain porous structures. We employed a 12 ms on and 3 ms off-pulse cycle on the SPS machine with a characteristic time of 3.3 ms for each single pulse.

**Figure 1:**
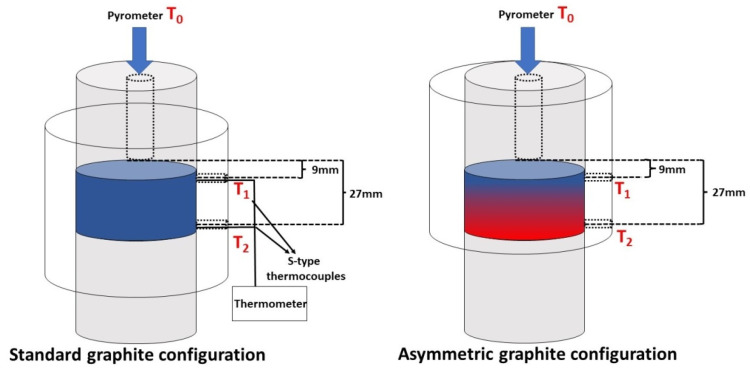
A schematic illustration of the graphite tool arrangement, as well as the positions of the sample in SPS graphite dyes.

To establish a porosity gradient structure, we employed an asymmetric graphite configuration, in which the samples were placed in an asymmetric position (ASY) with respect to the graphite mould. This asymmetric arrangement generated a temperature gradient within the sample. Sintering tests were also carried out with standard graphite configurations (STD) (the starting powders placed in the middle of the graphite mould), which serves as a reference material. To analyse the temperature gradients in the samples, we inserted two S-type thermocouples into the wall of the graphite tools both at the top (T_1_) and bottom (T_2_) parts of the samples, as shown in Figure 1. We used the temperature set for SPS as T_0_, which was controlled by the SPS pyrometer.

### 2.4. Characterization of Fibrous and Granular ZTA Ceramic Composites

The relative density of the sintered specimens was determined with the Archimedes method from which the porosity of the samples was calculated.

We analysed the phase composition and determined the structural characteristics of the as-prepared samples by XRD analysis (Philips PW 1830, Eindhoven, The Netherlands) using Cu Kα radiation. The XRD tests were carried out in the range of 20–70° 2θ in step-scanning mode, with a step size of 0.04°, and acquisition time of 1 s per angle. This analysis was performed on the as-synthetized ceramic fibres and on both sides of the sintered ceramic bodies.

The cross-section of the fractured samples was analysed by SEM (Zeiss EVO40, Zeiss, Jenna, Germany) with the use of a secondary electron detector (SE) in various magnifications and with an accelerating voltage of 20 kV. The samples were also examined with a backscattered electron (BSE) detector; the images were acquired at 3 and 10 kV by SEM (JSM-IT700HR, JEOL, Tokyo, Japan). Mechanical features were characterized by hardness and compressive strength. Vickers hardness was measured with the use of depth-sensing micro-indentation tests, with a CSM2008 instrument (Peseux, Switzerland) provided with a Vickers indenter tip, according to the standard of ISO 6507. The surfaces of the top and bottom sides of the samples were cut and polished prior to the tests. During each test, the load was held for 15 s with a force of 983 mN. Hardness was given as the mean value of five tests performed on the top and bottom parts of the ceramic bodies. For each test, the load–penetration depth curve was automatically plotted. Vickers hardness was calculated from the unloaded area of the load–depth curve.

Uniaxial compression testing was conducted at room temperature with an Instron 5566 tester, applying a constant crosshead speed of 0.5 mm/min. Strength was measured on three specimens for each specimen type. The dimensions of the specimens were 7 mm × 7 mm × ~4 mm.

To anticipate the possibility of using ceramics with gradient porosity, we impregnated the pore structure with nano-HAp. During the impregnation process, the porous ZTA composites were infused with the unfiltered, aqueous HAp slurry under vacuum for 3 h and then dried at 105 °C. The amount of absorbed HAp was determined by demanding the change in weight of the composites before and after impregnation. The composition of absorbed HAp and its position in the pore structure of the ZTA composites was examined by SEM-EDX analysis.

## 3. Result and Discussion

### 3.1. Temperature Gradient during the SPS Process

**Figure 2:**
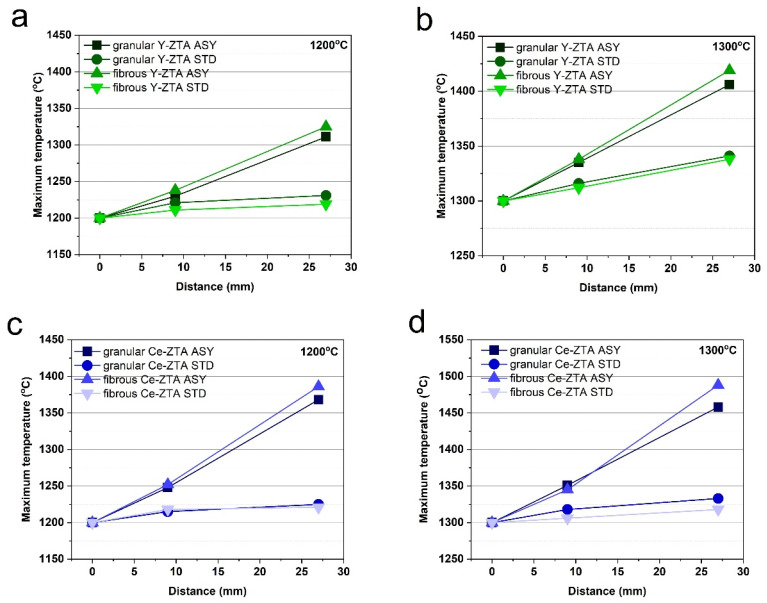
(**a**,**b**) (Y-ZTA) and (**c**,**d**) (Ce-ZTA) shows the maximum temperatures measured at three measurement spots (T_0_, T_1_ and T_2_) in the graphite mould for various sintering temperatures and starting materials. The ■ and ● symbols indicate the granular samples made in ASY and STD configurations, respectively, while ▲ and ▼ symbols belong to the fibrous samples made in ASY and STD configurations, respectively.

Figure 2 shows the maximum temperatures at several measurement spots along the graphite mould for the various test conditions (sintering temperature and mould configuration) and starting materials. The zero (starting) point is the top of the graphite mould, where T_0_ was measured by a pyrometer. Another two measurement spots (T_1_ and T_2_) were fixed at 9 and 27 mm away from T_0_ (Figure 1). This allows comparison of the temperature gradients in different tests, represented by the slope of the lines. The diagrams show that both the fibrous and granular ceramic bodies sintered in ASY configuration had a temperature gradient.

The temperatures of the top and bottom side of the samples are not considerably different for the STD configuration. The difference typically increases at higher sintering temperatures, as was also found in previous papers [21,22,23]. However, the maximum ΔT (ΔT = T_2_ − T_1_) was only 25 and 26 °C at 1300 °C sintering temperature for the fibrous and granular Y-ZTA samples, respectively.

In contrast, for the samples made in ASY configuration, there was a relatively large ΔT, which increased further with increasing sintering temperature. The maximum temperatures between the T_1_ and T_2_ spots for the ASY samples were more than 70 °C and 100 °C for the Y-ZTA and Ce-ZTA samples, respectively. Accordingly, significant ΔT was formed in the samples.

The morphology of the starting material had no significant influence on ΔT (Figure 2). ΔT is similar for the fibrous and granular samples with the same composition. The maximum temperature difference between granular and fibrous samples varied between 6 and 36 °C, depending on the applied sintering temperatures as well as the composition. However, in all cases, samples with a fibre morphology showed a higher temperature gradient. We assume that granular samples have far more contact points among particles than fibres, which cause more discharges among the spherical particles during SPS, which in turn results in a smaller temperature difference inside the samples. However, few studies focus on that issue.

As Figure 2 shows, ΔT varies based on the composition of the samples. ΔT was typically 55–80% higher for Ce-ZTA samples than for Y-ZTA samples, reaching an absolute value of 143 °C at a sintering temperature of 1300 °C. This relatively high difference in ΔT can be explained by the various substitutional defects in the structure of ZrO_2_ stabilized by ceria or yttria [31], which affect the thermal features of the materials. In contrast, in STD configurations, there were no significant differences in ΔT.

### 3.2. Porosity of the Ceramic Composites

**Figure 3:**
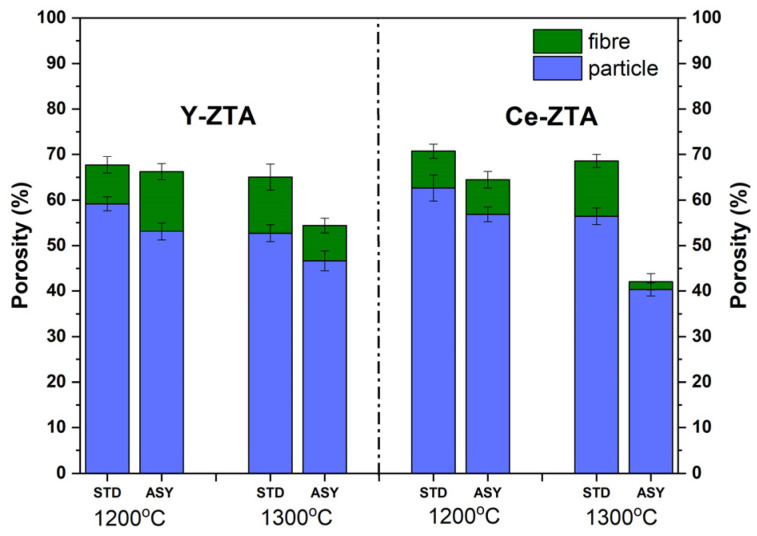
Porosity of the fibrous and granular Y-ZTA and Ce-ZTA samples sintered at different temperatures in STD and ASY configuration.

The total porosity of the sintered samples varied between 40 and 70%. As Figure 3. Shows, porosity shows a certain tendency with respect to composition, sintering temperature and the morphology of the starting material. Porosity decreased when sintering temperature was increased from 1200 °C to 1300 °C, which seems evident considering that a higher temperature facilitates consolidation. The samples sintered in STD configuration typically have higher porosity than samples sintered in ASY configuration. It can also be explained by the higher temperature typical for ASY configuration.

Fibrous samples for a given temperature, graphite configuration and composition exhibit 10–20% higher porosity than granular samples. This is probably due to the difference in compaction of materials with different morphologies during SPS sintering. Spherical nanoparticles can be compacted more efficiently and, as a consequence, more contact points are formed among the particular grains compared to fibrous materials.

### 3.3. Microstructure Analysis of the Composites

The phase compositions of sintered ceramic bodies were determined by XRD analysis. Phase analysis was performed on both sides of the sintered samples, in order to reveal the effect of the temperature differences on phase composition.

Regardless of morphology, the phase composition of the starting materials was the same for all samples (Figure S3). In addition to the most stable α-Al_2_O_3_, the initial fibrous and granular ceramics also contain γ- and δ-Al_2_O_3_ polymorphic phases, despite the calcination temperature of the precursors was over the formation temperature of α-Al_2_O_3_. This can be attributed to ZrO_2_ content, which can increase the phase transition temperature of polymorphic Al_2_O_3_ phases [32].

**Figure 4:**
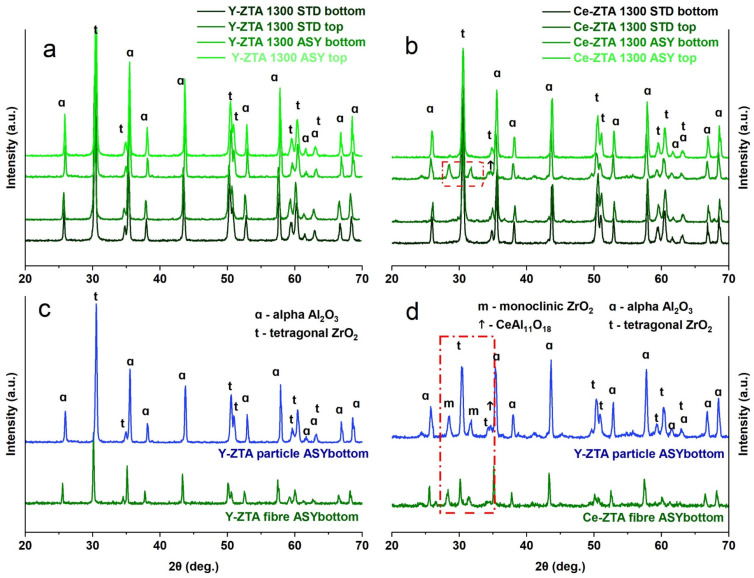
Phase composition of (**a**) Y-ZTA (**b**) Ce-ZTA granular samples fabricated in STD and ASY modes at 1300 °C. (**c**) and (**d**) XRD pattern of the Y-ZTA and Ce-ZTA sample, respectively, fabricated in ASY focusing on the phase differences between granular and fibrous samples at the bottom side.

After the SPS treatments, we only detected the α-Al_2_O_3_ polymorph. The granular samples are not significantly different from the fibrous samples (Figure 4). Regardless of graphite configuration, there was only α-Al_2_O_3_ and tetragonal ZrO_2_ (t-ZrO_2_) on both sides of the Y-ZTA samples sintered at either 1200 °C or 1300 °C. However, the phase composition of the granular Ce-ZTA sample changed at 1300 °C sintering temperatures, similarly to the fibrous samples (Figure 4b,d). The increased local temperature at the bottom side of the Ce-ZTA samples resulted in tetragonal to monoclinic ZrO_2_ phase transformation in ca. 7% (phase transformation are indicated by the red dashed box in Figure 4b,d). Also, CeAl_11_O_18_ also appears in a minor amount among the phases at the bottom side, as well as in the fibrous sample (Figure 4d), due to the locally higher temperature. The reason for the formation of CeAl_11_O_18_ was discussed in [22].

**Figure 5:**
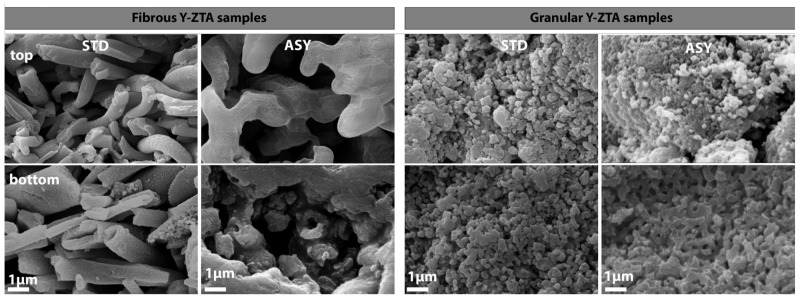
SEM images of fibrous and granular Y-ZTA samples fabricated in ASY and STD configuration at 1300 °C.

**Figure 6:**
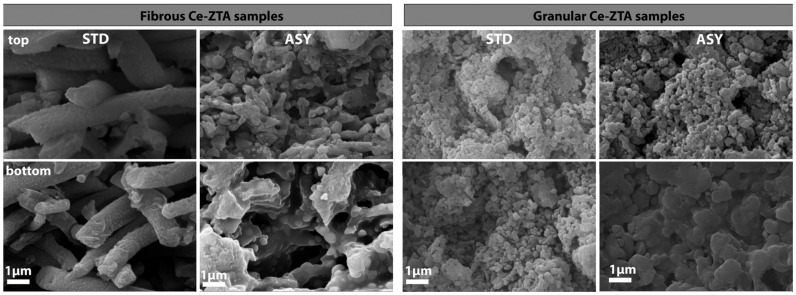
SEM images of fibrous and granular Ce-ZTA samples fabricated in ASY and STD configuration at 1300 °C.

SEM examinations were performed along the cross section of the fibrous and granular samples prepared in ASY and STD configurations. Figure 5 and Figure 6 show SEM images of the Y-ZTA and Ce-ZTA samples, respectively, and separately present the fibrous and granular samples sintered at 1300 °C.

The difference is clearly visible in the microstructure of the samples sintered in different graphite configurations in terms of gradient structure and density. The gradient microstructure is clearly apparent for the ASY samples, regardless of the fibrous or granular morphology of the sample, while the top part of the ASY samples consists of fine particles or fibres with substantial porosity—especially for the granular samples—the bottom part consists of coarser grains and fibres, leaving less room between them, which results in decreased porosity. The extent of grain coarsening, however, is smaller for the granular Y-ZTA sample than in the Ce-ZTA sample, leaving smaller pores (100–150 nm) in the structure, which resulted in higher porosity. This can be ascribed to the much higher temperature of Ce-ZTA samples, especially in ASY configurations, compared to Y-ZTA samples. Consequently, the microstructure of the granular Ce-ZTA samples is almost pore-free at the lower part.

In contrast, the gradient microstructure failed to develop in STD samples, regardless of either initial morphology or composition: the microstructure is identical at the top and bottom side of the samples (Figure 5 and Figure 6), i.e., grain size and pore structure are almost the same throughout the sample. However, pore structure varies even in STD configuration, depending on the morphology of the initial ceramics. The porosity of the fibrous samples is much higher compared to the granular samples, due to the lower sintering rate of the fibres. As for the granular samples, the enlarged grains with extended connecting areas resulted in a more compacted structure with less interconnected porosity.

SEM analysis also revealed the formation of an intra-intergranular ZrO_2_-Al_2_O_3_ structure, similarly to the fibrous samples. The high-resolution SEM micrographs (Figures 9 and 10) clearly show the intragranular position of ZrO_2_ particles in the Al_2_O_3_ grains for the granular samples, as well. However, a comparison of the granular and fibrous samples show that ZrO_2_ grain coarsening is less intensive for fibrous Y-ZTA samples, and ZrO_2_ grains typically remain in an intragranular position compared to Ce-ZTA samples.

### 3.4. Mechanical Properties of the Composites

**Figure 7:**
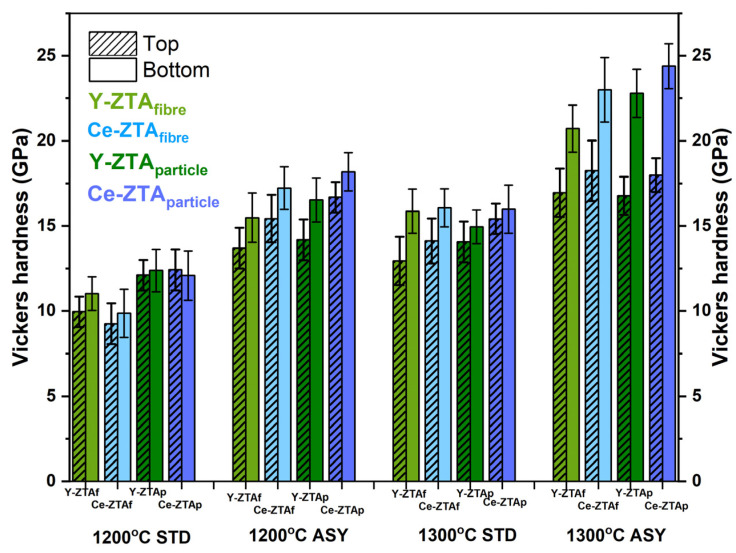
HV values of the fibrous and granular Y-ZTA (green) and Ce-ZTA (blue) samples sintered under various sintering conditions.

Mechanical properties of the composites were investigated in terms of microhardness and compressive strength determined by microindentation and compression tests, respectively. Vickers hardness (HV) tests were performed on both sides of the samples because of the gradient microstructure of the ASY samples. Figure 7 shows the Vickers hardness of the samples sintered under various conditions.

(a) *Effect of the graphite configuration*

There is a definite but almost negligible (typically less than 1 GPa) difference in Vickers hardness between the two sides of the samples fabricated in the STD configuration. As expected, the higher sintering temperature resulted in greater hardness.

In contrast, for ASY samples, the temperature difference between the top and bottom sides clearly affected hardness. The bottom of the samples with a higher temperature typically had significantly higher hardness than their top side. Here too, the higher sintering temperature led to greater hardness. In addition, the hardness difference between the two sides of the samples increased even larger, reaching 8–9 GPa. This can be attributed to the gradient porosity along the cross-section. As the SEM images show (Figure 5 and Figure 6), porosity in the top side of the ASY samples was much higher than in their bottom side.

(b) *Effect of the various stabilisation agents on hardness*

The ZTA samples with CeO_2_ content exhibit greater hardness than the composites stabilized with Y_2_O_3_, even though we observed a tetragonal to monoclinic ZrO_2_ transformation at the bottom side of the Ce-ZTA samples due to the higher local temperature there.

However, we assume that hardness can be better tailored by the pore structure than by the composition. The Y-ZTA samples tend to preserve their fine structure regardless of the high local temperature, and contain smaller Y_2_O_3_–stabilized ZrO_2_ grains in intergranular positions within the Al_2_O_3_ grains. However, Y-ZTA samples still exhibit lower hardness than the Ce-ZTA samples. As the SEM micrographs also proved, grain coarsening in Ce-ZTA samples is more significant and the proportion of intragranular grains is also lower (Figure 10), which generates a more stable pore structure with greater hardness.

(c) *The effect of initial morphology on hardness*

Morphology also affects hardness (Figure 7). Samples with granular morphology typically exhibit greater hardness than samples with a fibrous structure under the same sintering conditions. Although the difference is obvious, it is not significant, because other parameters also have an influence on hardness.

The higher hardness of the granular sample can be ascribed to the significantly different porosity and pore structure of the sintered sample. This is supported by the higher hardness of the bottom side of the samples, which usually have lower porosity, especially in the ASY configuration. In the case of granular samples, larger connecting areas of the initial particles can be observed due to their spherical shape, which results in smaller porosity, but at same time, provides a more stable and compacted structure. In the case of fibrous samples, porosity was higher and shows a more extended interconnected pore structure with large pores. Despite the relatively high porosity, the hardness of both morphology series is higher than hardness in the relevant literature [14,33], probably due to the very fine intra-intergranular structure of the composites, which is favourable in terms of hardness.

**Figure 8:**
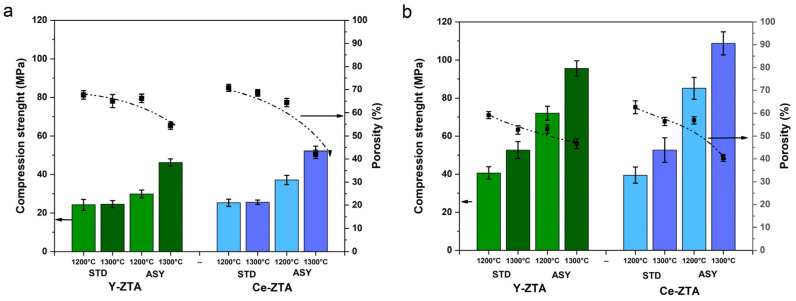
Compression strength and the related porosity values of (**a**) the fibrous and (**b**) granular Y-ZTA (green) and Ce-ZTA (blue) samples at various sintering conditions.

Figure 8a,b show the compressive strength, with the porosity values of fibrous and granulate ZTA ceramics, respectively, depending on sintering temperature and graphite configuration.

Strength shows a trend similar to hardness. As sintering temperature increased, the strength of the samples improved for both compositions. Furthermore, the influence of the ASY configuration is more obvious due to the even higher temperature than the set temperature. While there is no or negligible difference between the Y-ZTA and Ce-ZTA samples in the STD configuration, there is a significant difference between the ASY samples. This can also be attributed to the strong correlations between mechanical properties and the microstructure that developed, as discussed above.

Figure 8 shows that morphological differences still have the most significant effect on the strength of the samples. The granular ZTA samples (Figure 8b) have much greater compression strength than the fibrous samples (Figure 8a). For the granular and fibrous samples, the trend is very similar based on composition, sintering temperature, and graphite configuration, but granular samples had ~50% higher strength. Higher compression strength can be attributed to the more stable and compacted pore structure in the granular samples.

The highest compression strength was 108 ± 6.1 MPa (granular Ce-ZTA sample sintered in ASY at 1300 °C), even though, in this case, the detected T_1_ and T_2_ temperatures as well as ∆T were smaller than for the fibrous Ce-ZTA sample prepared under the same conditions. The same trend can be observed for the granular Y-ZTA sample, whose compression strength was 95 ± 4.1 MPa, while the fibrous Y-ZTA reached only half that strength (46 ± 1.9 Mpa).

The higher local temperature and, therefore, the higher ∆T is only one factor. The effect of morphology is also a significant factor contributing to an enhanced mechanical strength. Yet, it cannot be unequivocally stated that the granular morphology is better because of the smaller porosity (around ~40%) of these samples, as shown in Figure 8.

Our results show that a combination of ceramics with mixed morphologies would also be appropriate, e.g., in a layered arrangement. For increased porosity, ceramics fibres are more suitable, while granular ceramics are better for an enhanced strength.

### 3.5. Improving the Biocompatibility of the Composites by Infiltration with HAp

We also investigated the potential application of such ZTA ceramics with a high porosity gradient as well as enhanced strength. ZTA is also an important bioceramic; therefore, we integrated bioactive HAp material into the pore structure, since deposited HAp inside the pores is known to possess bone-bonding properties [33].

We impregnated HAp slurry into fibrous and granular ZTA ceramic samples sintered in the ASY configuration at 1300 °C, which reached the most optimal properties in terms of gradient porosity as well as mechanical properties (Table 1).

As shown in Table 1, the fibrous and granular Y-ZTA samples have a higher porosity as compared to the Ce-ZTA samples. Accordingly, the amount of absorbed HAp was also higher in this case. The impregnated HAp was around 1% of the total weight of the sample for Y-ZTA samples and ca. 0.6% for the Ce-ZTA samples. The absorption capacity of Ce-ZA composites is much lower, presumably due to their smaller porosity. The composites with fibrous morphology are more effective than granular composites. This result can be attributed to the bimodal pore structure of ceramics with fibrous morphology.

**Figure 9:**
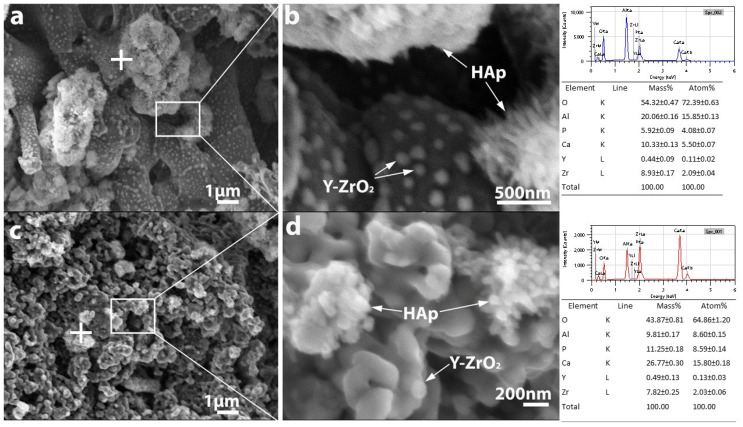
The fracture surface of fibrous (**a**,**b**), and granular (**c**,**d**) Y-ZTA ceramics in lower and higher magnification impregnated with HAp. The white cross on the figure a and c shows the location of the EDX analysis of both samples.

**Figure 10:**
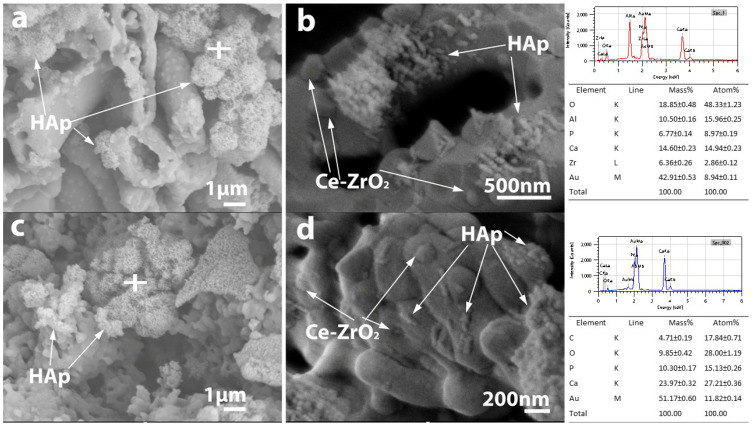
The fracture surface of fibrous (**a**,**b**), and granular (**c**,**d**) Ce-ZTA ceramics in lower and higher magnification impregnated with HAp. The white cross on the figure a and c shows the location of the EDX analysis of both samples.

HAp absorption of the ceramics was also investigated by high-resolution SEM and EDX analysis (Figure 9 and Figure 10). (Au and C appearing in the EDX spectra originate from the conductive thin film usually applied for SEM analysis).

The SEM micrographs confirmed the presence of HAp in the pore structure inside the porous ZTA composites, while the EDX analysis confirmed that the Ca/P atomic ratio of the absorbed HAp is appropriate (Figure 9 and Figure 10). High-resolution SEM images revealed that HAp appears in two different morphologies (Figure 9b): it appears in the form of small whiskers and as spherical particles as well with ~100 and 50 nm in size, respectively. Based on the SEM analysis, HAp agglomerates that are several micrometres in size are able to pass into the pore structure of the samples with either fibrous or granular morphologies, regardless of composition. In addition to agglomerates, individual nanosized HAp particles also appear. Figure 10b shows that in the case of the bimodal pore structure characteristic of fibrous samples (large pores between the fibres and small pores inside the fibres), HAp can integrate into the fibres, as well.

Our results suggest that such ZTA ceramic bodies with gradient porosity are suitable for biomedical use, and biocompatibility can be enhanced by integrating HAp into the pore system.

## 4. Conclusions

In this paper, we studied highly porous Y-ZTA and Ce-ZTA ceramic composites with improved mechanical properties, comparing the effect of the nanofibrous and nanogranular morphologies of the starting materials. A gradient structure was produced in the ceramic samples by creating a temperature gradient during sintering with the use of asymmetric SPS graphite tool arrangements. As a reference, ceramic bodies with the same composition and morphology were also fabricated with a standard (normal) SPS graphite configuration. We investigated the effect of the morphology of the starting materials on the temperature gradient during sintering, as well as on the microstructure and mechanical behaviour of the sintered ceramics. Considering possible practical applications of the porous ceramic materials, we impregnated nanosized HAp-slurry into the ZTA ceramics and investigated the as-formed porous microstructure.

Our major findings can be summarized as follows:

(1) There was a considerably larger temperature difference for both the granular and fibrous samples made in the ASY graphite configuration than for samples produced in the STD configuration. In addition to graphite configuration, the stabilizing agent (ceria) also affects ΔT. For the Ce-ZTA samples, the temperature differences were 50–70% higher as compared to Y-ZTA samples in the ASY configuration.
(2) The pore structure of the ceramic body can be tailored by varying the morphology of the precursor or the graphite configuration. While the total porosity of the composites varied in the range of 40 to 65%, the fibrous structure resulted in a significantly and typically 10–20% higher porosity than the granular structure, independent of any other conditions. The significant temperature gradients during the sintering process in the ASY graphite configuration led to a porosity gradient along the cross-section of the samples. Owing to the lack of temperature gradient in the STD configuration, the microstructure of those samples was homogenous.
(3) The gradient porosity in both fibrous and granular ZTA samples allows facile infiltration of bioactive Hap slurry into the pore structure, although fibrous ZTA samples are more suitable to absorb nanosized Hap due to their higher porosity and bimodal pore structure.
(4) The mechanical properties of the ZTA composites are affected by several factors, including the morphology of the starting materials, graphite configuration, and the doping agent, as well.
  (a) Closely related to the developing microstructure of the composites, the hardness of the two opposite sides of each composite sintered in the ASY configuration differed considerably. This difference reached as much as 9 GPa for the granular ZTA samples sintered at 1300 °C. In contrast, samples fabricated in the STD configuration had almost the same hardness on opposite sides. Considering the effect of morphology on hardness, the samples with granular morphology exhibit typically higher hardness than samples with a fibrous structure under the same sintering conditions. Using CeO_2_ instead of Y_2_O_3_ as the stabilizing agent for ZTA also increases hardness, probably because of the more compacted microstructure of Ce-ZTA with less porosity.
  (b) The compression strength of the composites is affected by almost each factor. The influence of various factors on compression strength decreased in the order of morphology, graphite configuration, sintering temperature, and stabilizing element. The granular Ce-ZTA composite sintered in ASY at 1300 °C had the highest compression strength (108 ± 6.1 MPa).

Our results suggest that the combination of fibrous and granular ZTA in a layered arrangement would be the most appropriate configuration to obtain a ceramic with high porosity, enhanced absorption capacity and improved mechanical strength.

## Figures and Tables

**Table 1:** Porosity of the sintered samples used for HAp impregnation tests and the amount of absorbed HAp.

Samples	Porosity (%)	Amount of Absorbed Hap (%)
fibrous Y-ZTA	54.4	1.2
granular Y-ZTA	46.7	1.1
fibrous Ce-ZTA	42.0	0.7
granular Ce-ZTA	40.3	0.5

## Data Availability

The data that support the findings of this study are available from the corresponding author.

## Supplementary Materials

The following supporting information can be downloaded at: https://www.mdpi.com/article/10.3390/mi14091681/s1, Figure S1: Morphology of the granular Y-ZTA (a) and Ce-ZTA (b) composite ceramics; Figure S2: Phase composition and structure of HAp; Figure S3: Phase composition of fibrous and granular reference ZTA samples.
