# Supplementary material for: Zirconia-Toughened Alumina (ZTA) Nanoceramics with a Gradient Microstructure: A Comparative Study of ZTA Ceramics with Fibrous and Granular Morphology
Source: Micromachines (Basel). 2023 Aug 28;14(9):1681. doi: 10.3390/mi14091681 (PMC10534487; doi:10.3390/mi14091681)
Supplement: Supplementary file 1 [file micromachines-14-01681-s001.zip › micromachines-2561340-supplementary.pdf]

micromachines

MDPI

Supplementary Materials

Zirconia-Toughened Alumina (ZTA) Nanoceramics with A Gradient Microstructure: A Comparative Study of ZTA Ceramics with Fibrous and Granular Morphology

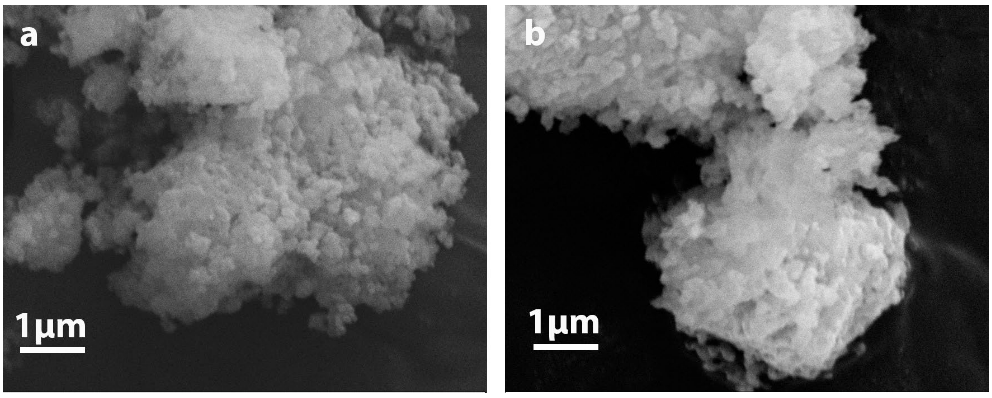

Figure S1. Morphology of the granular Y-ZTA (a) and Ce-ZTA (b) composite ceramics.

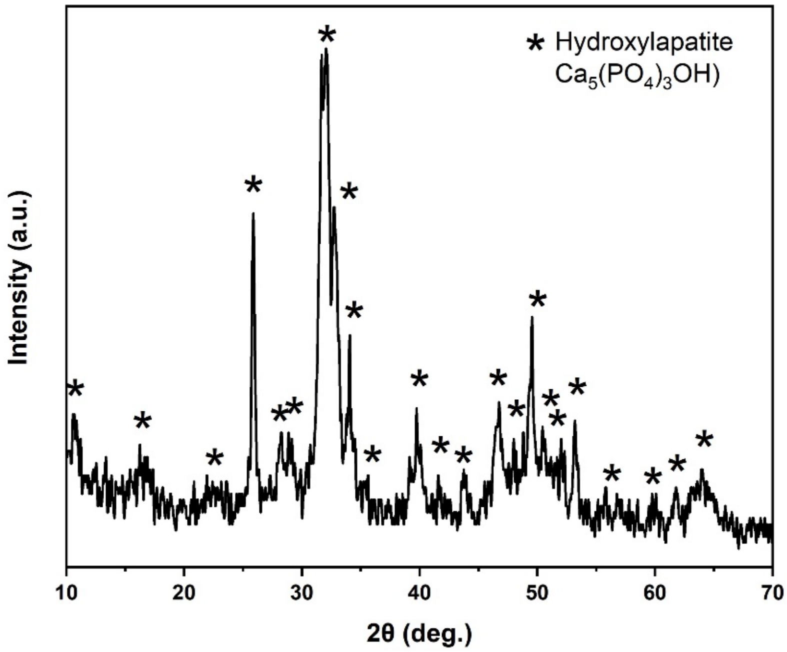

Figure S2. Phase composition and structure of HAp.

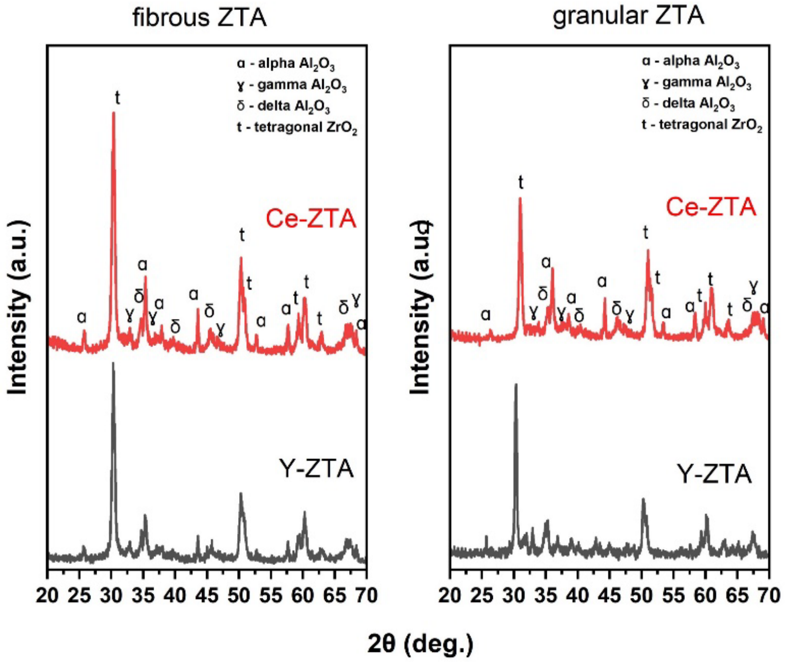

Figure S3. Phase composition of fibrous and granular reference ZTA samples.
